# Interactions between Exposure to Environmental Polycyclic Aromatic Hydrocarbons and DNA Repair Gene Polymorphisms on Bulky DNA Adducts in Human Sperm

**DOI:** 10.1371/journal.pone.0013145

**Published:** 2010-10-05

**Authors:** Guixiang Ji, Aihua Gu, Yong Zhou, Xiangguo Shi, Yankai Xia, Yan Long, Ling Song, Shoulin Wang, Xinru Wang

**Affiliations:** 1 Key Laboratory of Reproductive Medicine, School of Public Health, Institute of Toxicology, Nanjing Medical University, Nanjing, China; 2 Massachusetts General Hospital, Harvard Medical School, Boston, Massachusetts, United States of America; Institute of Zoology, Chinese Academy of Sciences, China

## Abstract

**Background:**

Nucleotide excision repair (NER) and base excision repair (BER) are the primary mechanisms for repair of bulky adducts caused by chemical agents, such as PAHs. It is expected that polymorphisms in NER or BER genes may modulate individual susceptibility to PAHs exposure. Here, we evaluate the effects of PAHs exposure and polymorphisms in NER and BER pathway, alone or combined, on polycyclic aromatic hydrocarbon-DNA (PAH–DNA) adducts in human sperm.

**Methodology/Principal Findings:**

Sperm PAH-DNA adducts were measured by immunofluorescent assay using flow cytometry in a sample of 465 infertile adults. Polymorphisms of *XPA*, *XPD*, *ERCC1*, *XPF*, and *XRCC1* were determined by polymerase chain reaction (PCR) and restriction fragment length polymorphism (RFLP) techniques. The PAHs exposure was detected as urinary 1-hydroxypyrene (1-OHP) levels. In multivariate models adjusted for potential confounders, we observed that *XRCC1* 5′pUTR -T/C, Arg194Trp, Arg399Gln polymorphisms were associated with increased sperm adduct levels. Furthermore, the stratified analysis indicated that adverse effects of *XRCC1* Arg194Trp, Arg399Gln polymorphisms on PAH-DNA adducts were detected only in the high PAHs exposure group.

**Conclusions/Significance:**

These findings provided the first evidence that polymorphisms of *XRCC1* may modify sperm PAH-DNA adduct levels and may be useful biomarkers to identify individuals susceptible to DNA damage resulting from PAHs exposure.

## Introduction

Over the last two decades, there has been a growing concern regarding the progressive decline in male fertility [1], [2]. Although possible reasons remain unclear, several studies have reported associations between exposure to some environmental chemicals and poor sperm function [3]–[7]. Recently, polycyclic aromatic hydrocarbons (PAHs) are received more and more attentions because they are widespread and prone to attack DNA by forming PAH-DNA adducts [8], [9].

In China, due to the conventional eating habits that involve heavily fried, roasted, or grilled foods and the rapid increase of automobile and industrial production, the general population has more opportunities to be exposed to PAHs from multiple sources and routes compared to other nations. However, it is difficult to assess the exact exposure levels of PAHs from multiple routes. Recently, the urinary metabolite 1-hydroxypyrene (1-OHP) has been extensively characterized and may reflect internal PAHs exposure as a sensitive biomarker [10], [11]. Our previous studies among Chinese men have assessed the non-occupational exposure to PAHs by measuring 1-OHP concentrations in urine. Notably, it is found that the median concentration of CR-adjusted 1-OHP in our study is almost 16-fold higher than those in U.S. populations [6]. This result suggests that Chinese adult males are highly exposed to PAHs in the environment, and possess extremely high exposure levels, so the potential health hazard of PAHs deserves more attention in China.

PAHs derive their genotoxic and carcinogenic properties through their abilities to form PAH-DNA adducts that may be a potential source of transmissible prezygotic DNA damage in spermatozoa [12]. Although only limited studies have concerned DNA adducts in human sperm to date, it has been demonstrated that PAH-DNA adducts in sperm are correlated with abnormal sperm morphology, decreased sperm count, declined progressive motility and impaired fertilization during ICSI (intracytoplasmic sperm injection) [13]–[16]. These results suggest that DNA adducts in sperm can be applied as potential biomarkers in studies of human infertility.

To cope with the sperm DNA damage caused by genotoxic adverse factors, the human has developed defensive mechanisms including several DNA repair pathways that faithfully remove DNA lesions. Although the main pathway for removal of bulky DNA adducts is the nucleotide excision repair (NER), it has been shown that the base excision repair (BER) mechanism may also participate in adduct repair [17]. Recently, common genetic polymorphisms in DNA repair genes are hypothesized to result in reduced capabilities of DNA damage repair, and thus may be a useful biomarker to identify individuals sensitive to the environmental genotoxic chemical.

In the present study, we detected PAH-DNA adducts in ejaculated sperm of infertile adults environmentally exposed to low levels of PAHs, and evaluated their relation with functional SNPs in *XPA*, *ERCC1*, *XPD*, *XPF*, and *XRCC1*. Furthermore, we also addressed the interaction between these genetic polymorphisms and PAHs exposure on sperm PAH-DNA adducts levels. To the best of our knowledge, this is the first study that investigates the effects of low levels PAHs exposure and DNA repair polymorphism, alone or combined, on sperm PAH-DNA adducts.

## Results

**Figure 1:**
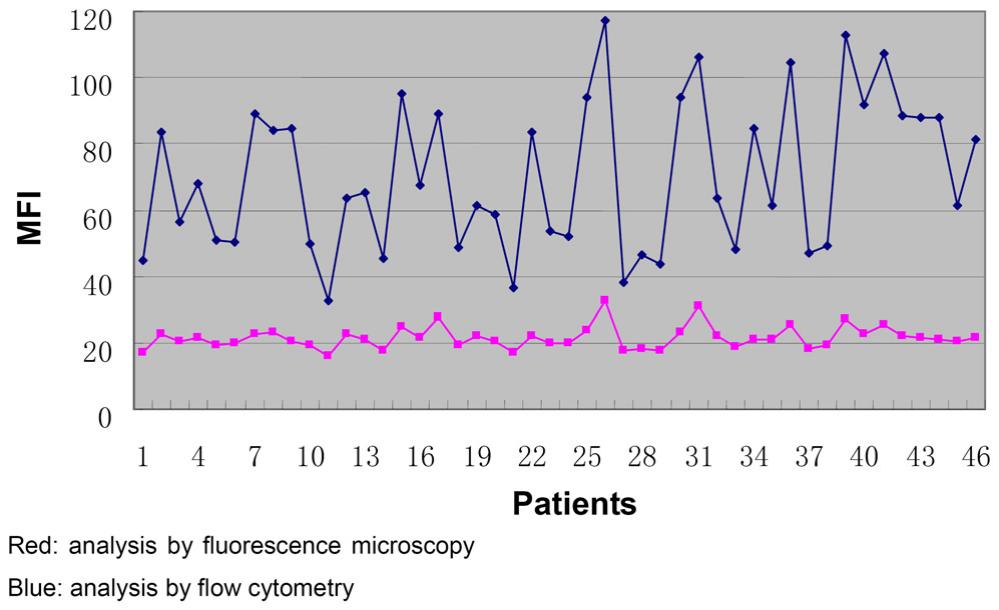
Comparison of immunofluorescence analysis by flow cytometry and fluorescence microscopy in 46 patients.

**Figure 2:**
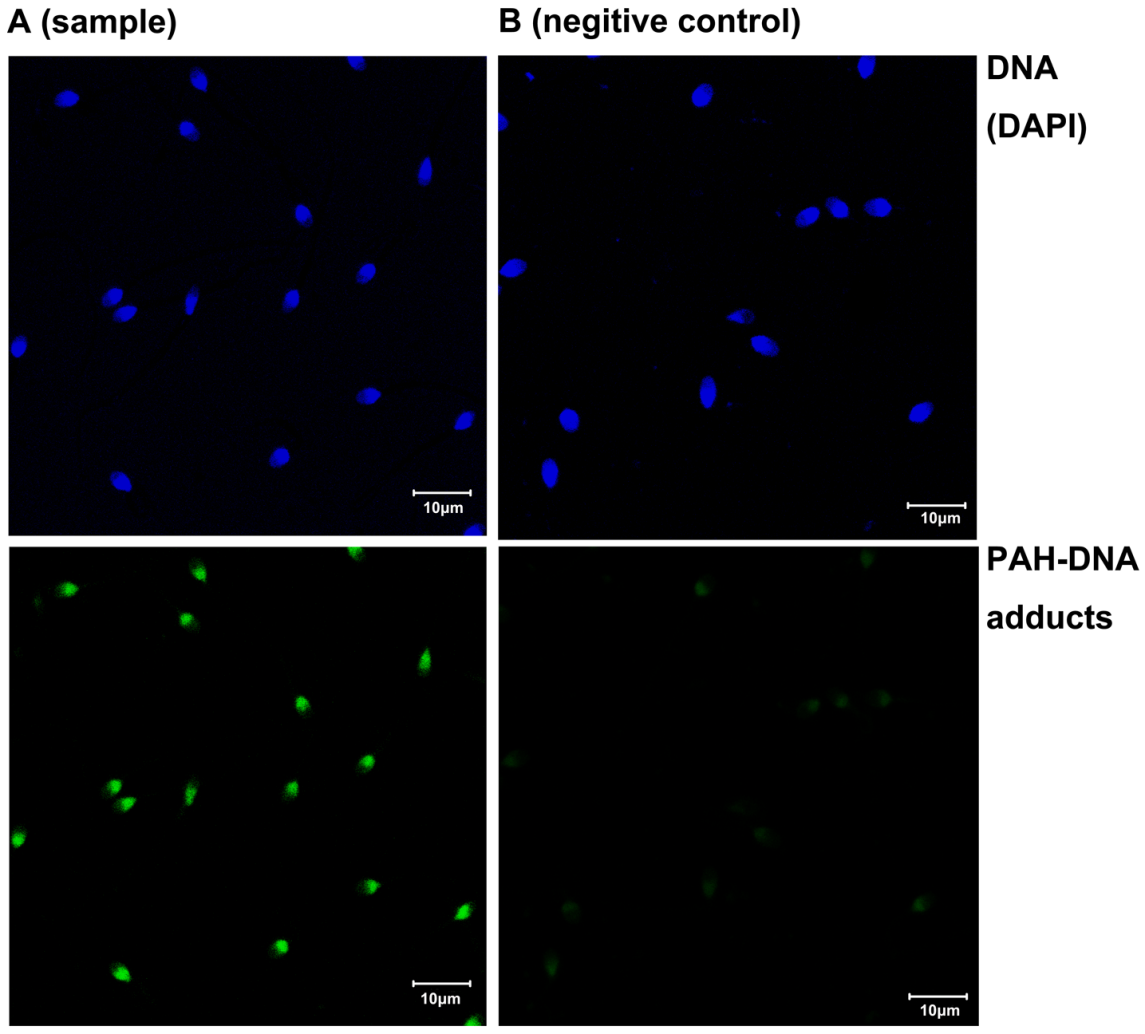
Sperm PAH-DNA adducts was detected by an immunofluorescence staining with FITC-labeled antibody (green), sperm DNA was counterstained with DAPI (blue). (A) A sample (B) Negative control untreated with 5D11 monoclonal antibody.

**Figure 3:**
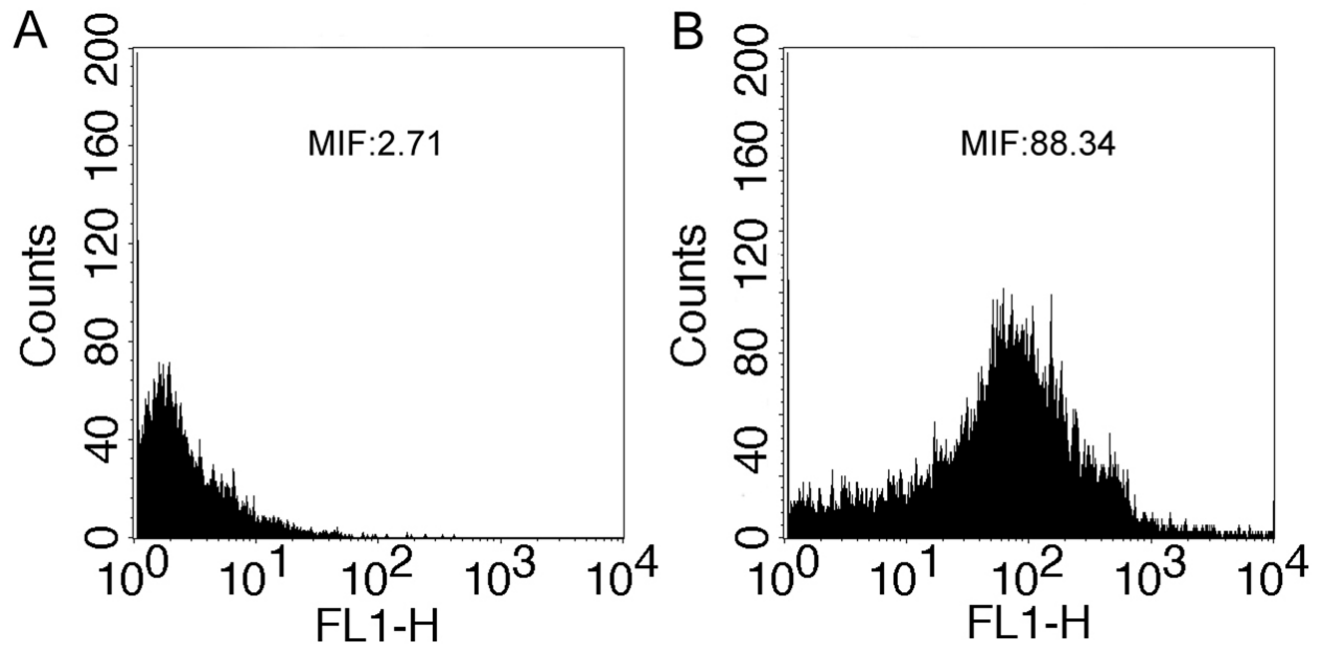
Detection of immunofluorescence staining by flow cytometry. Relative cell numbers (ordinate) and fluorescence intensity on a log10 scale (abscissa) are indicated. A negative control with fluorescence intensity (MIF) of 2.71% (A) and a sample with MFI of 88.34% (B) were illustrated.

To assess the efficiency and concordance of flow cytometry and fluorescence microscopy, we applied these two approaches in a total of 46 human sperm samples. A good correlation is detected between the results as measured by flow cytometry and fluorescence microscopy (**Figure 1**; r = 0.88, *P*<0.001). In Figure 2, representative immunofluorescent images of a negative control and a sample are shown. As can be seen clearly, the sample showed clear fluorescence signals of binding to PAH-DNA adducts (**Figure 2A**), whereas only minimal staining was observed in negative control (**Figure 2B**). With flow cytometry, the negative control showed low fluorescence (**Figure 3A**, MIF: 2.71) while the sample showed strong fluorescence (**Figure 3B**, MIF: 88.34). These results suggested that immunofluorescence method using BPDE-DNA (5D11) monoclonal antibody is useful for both in situ and flow cytometry detection of sperm PAH-DNA adducts. Compared to fluorescence microscopy, flow cytometry allows the measurement of PAH-DNA adducts at the single cell level, as well as automatic analysis of thousands of cells in a few seconds and therefore was used in this study.

**Figure 4:**
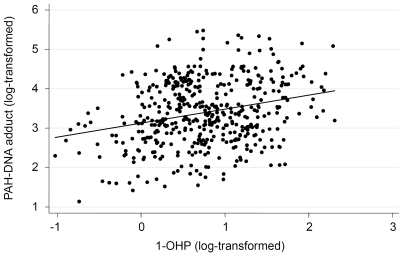
The correlation between the natural log transformed of the urinary 1-OHP and the natural log transformed of sperm PAH-DNA addcuts (r = 0.262, *P* = 0.003).

The distribution profiles of sperm PAH-DNA adducts and urine 1-OHP levels among 465 patients, as shown in **Figure S1**, **S2**, underwent natural logarithmic transformation for further association studies. The individual characteristics and the effects on sperm PAH-DNA adducts and urinary 1-OHP levels were exhibited in **Table 1**. Significantly increased levels of PAH-DNA adducts were observed in subjects with high levels of urinary 1-OHP, which was supported by the Pearson correlation analysis (r = 0.262, *P* = 0.003) (**Figure 4**). Moreover, we found that the dietary intake of PAH-rich meals significantly influenced sperm adduct levels. Subjects who consume PAH-rich meals ≥3 times/week had significantly higher adduct levels than those who did not (mean ± S.D., 3.62±0.94 versus 3.35±0.86; *P* = 0.043). Other characteristics, such as age, cigarette smoking, alcohol drinking, and area of residence appeared to have no obvious effects on sperm PAH-DNA adduct levels.

**Table 1:** Subject characteristics, urinary 1-OHP levels and sperm PAH-DNA adducts.

Variables	Subjects*N* (%)	PAH-DNA adducts^b^(Mean ± S.D.)	*p* c	1-OHP^b^(Mean ± S.D.)	*p* c
**Age (years)**					
<31	230 (49.5)	3.36±0.83	0.370	0.74±0.61	0.720
≥31	235 (50.5)	3.43±0.85		0.76±0.60	
**Smoking status**					
Never	173 (37.2)	3.35±0.80		0.70±0.58	
Current	249 (53.5)	3.41±0.81	0.452	0.77±0.60	0.600
Former	43 (9.2)	3.49±0.95	0.324	0.89±0.66	0.187
**Drinking status**					
Yes	73 (15.7)	3.33±0.92	0.570	0.74±0.50	0.794
No	392 (84.2)	3.39±0.81		0.76±0.62	
**Area of residence**					
Rural	198 (42.6)	3.45±0.88	0.239	0.74±0.58	0.655
Urban/suburban	267 (57.4)	3.36±0.76		0.77±0.62	
**Consumption of charcoaled food**					
0	298 (64.1)	3.35±0.86		0.72±0.59	
1–2/week	117 (25.2)	3.42±0.89	0.460	0.78±0.61	0.657
≥3/week	50 (10.8)	3.62±0.94	**0.043**	0.92±0.64	0.086
**Urinary 1-OHP (**µ**g/g of CR)** a **^, ^** b					
≤0.71	232 (50.0)	3.30±0.82	**0.010**	0.27±0.35	**<0.001**
>0.71	233 (50.0)	3.49±0.81		1.24±0.37	

a Urinary 1-OHP levels are creatinine (CR)-adjusted and expressed as µg/g of CR.

b natural log-transformed.

c Two-sided χ^2^ test.

The associations between DNA repair genetic polymorphisms and adduct levels were evaluated by multiple linear regression among 465 patients (**Table 2**). Given the sample size of our study, for all SNPs we assumed a dominant mode of inheritance. Compared with the *XRCC1* 5′UTR TT homozygotes, the *XRCC1* 5′UTR TC heterozygotes carriers showed significantly higher adduct levels (mean ± S.D., 3.35±0.82 versus 3.60±0.80, *P* = 0.012). Furthermore, we observed that individuals carried the *XRCC1*-194 T allele or *XRCC1*-399 AA homozygotes displayed markedly higher levels of PAH-DNA adducts, compared with the wild-type homozygote. However, no obvious associations were observed between the other DNA repair genetic polymorphisms and adduct levels in total sample.

**Table 2:** Effects of DNA repair genetic polymorphisms on sperm PAH-DNA adducts.

Genotype	Subjects	PAH-DNA adducts^a^		*P* b ^, ^ c
	*N* (%)	Mean ± S.D.	Range	
***XRCC1*** ** 5′UTR**				
TT	379 (81.5)	3.35±0.82	1.13–5.45	Reference
TC	84 (18.1)	3.60±0.80	1.65–5.48	**0.012**
CC	2 (0.4)	3.46±0.77	2.92–4.01	0.844
***XRCC1*** ** Arg194Trp**				
CC	229 (49.2)	3.29±0.82	1.13– 5.48	Reference
CT	197 (42.4)	3.46±0.80	1.41–5.33	**0.034**
TT	39 (8.4)	3.71±0.82	1.75–5.45	**0.003**
***XRCC1*** ** Arg280His**				
GG	375 (80.7)	3.40±0.83	1.13–5.48	Reference
GA	83 (17.8)	3.37±0.77	1.77–4.87	0.738
AA	7 (1.5)	3.29±0.87	2.38–4.44	0.725
***XRCC1*** ** Arg399Gln**				
GG	239 (51.4)	3.37±0.78	1.52–5.48	Reference
GA	184 (39.6)	3.36±0.83	1.13–5.36	0.922
AA	42 (9.0)	3.69±0.94	1.54–5.45	**0.018**
***XPA*** ** 5′UTR**				
GG	115 (24.7)	3.35±0.84	1.41–5.45	Reference
GA	246 (52.9)	3.40±0.85	1.13–5.48	0.611
AA	104 (22.4)	3.45±0.87	1.52–5.08	0.390
***XPD*** ** Lys751Gln**				
GG	400 (86.0)	3.40±0.81	1.41–5.48	Reference
GA + AA	65 (14.0)	3.36±0.88	1.13–5.36	0.837
***ERCC1*** ** 3′UTR**				
GG	209 (44.9)	3.35±0.80	1.61–5.45	Reference
GA	212 (45.6)	3.43±0.82	1.13–5.48	0.325
AA	44 (9.5)	3.47±0.95	1.41–5.28	0.390
***XPF*** ** Ser835Ser**				
CC	248 (53.3)	3.36±0.82	1.13–5.45	Reference
CT	182 (39.1)	3.45±0.79	1.61–5.36	0.304
TT	35 (7.5)	3.37±0.93	1.61–5.48	0.971

a natural log-transformed.

b Two-sided χ^2^ test.

c Adjusted for consumption of charcoaled food and urinary 1-OHP levels.

**Figure 5:**
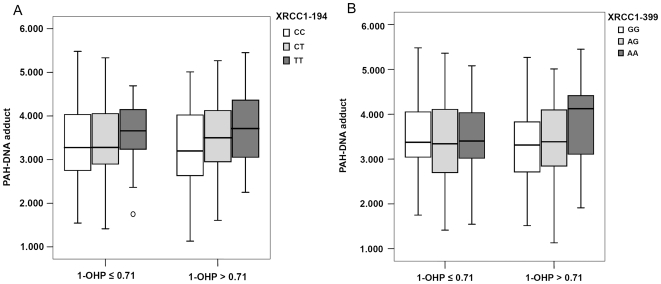
Sperm PAH-DNA adducts and effects of *XRCC1* Arg194Trp, Arg399Gln polymorphisms stratified by urinary 1-OHP levels.

For a better understanding of potential effects of environmental and genetic factors on the formation of sperm adducts, we examined whether there existed interactions between DNA repair genetic polymorphisms and PAHs exposure. Exposure variables were dichotomized into a low- and a high-exposure group by the urinary 1-OHP levels median value (natural log transformed: 0.71 µg/g of CR). Significantly higher levels of adducts were detected in carriers of the *XRCC1*-194 T allele compared with the wild-type homozygotes only in high-exposure group (**Table3**; **Figure 5**). Similar result was observed for the *XRCC1* Arg399Gln polymorphisms. These results indicated a joint effect between DNA repair genetic polymorphisms and PAHs exposure on susceptibility to sperm PAH-DNA adducts.

**Table 3:** Associations between DNA repair polymorphisms and sperm PAH-DNA adducts stratified by urinary 1-OHP levels.

Genotypes	Low-exposure group		High-exposure group	
	*N* (Mean ± S.D.)^a^	*P* b ^, ^ c	*N* (Mean ± S.D.)^a^	*P* b ^, ^ c
***XRCC1*** ** 5′UTR**				
TT	190 (3.35±0.82)	Ref	189 (3.35±0.77)	Ref
TC + CC	42 (3.37±0.78)	0.742	44 (3.65±0.98)	0.166
***XRCC1*** ** Arg194Trp**				
CC	125 (3.36±0.80)	Ref	104 (3.20±0.83)	Ref
CT	91 (3.38±0.84)	0.873	106 (3.52±0.77)	**0.004**
TT	16 (3.64±0.78)	0.190	23 (3.76±0.85)	**0.003**
***XRCC1*** ** Arg280His**				
GG	186 (3.39±0.83)	Ref	189 (3.41±0.83)	Ref
GA	44 (3.39±0.76)	0.971	39 (3.35±0.79)	0.661
AA	2 (2.80±0.59)	0.310	5 (3.49±0.94)	0.840
***XRCC1*** ** Arg399Gln**				
GG	116 (3.44±0.79)	Ref	123 (3.30±0.76)	Ref
GA	98 (3.32±0.84)	0.265	86 (3.41±0.83)	0.328
AA	18 (3.41±0.86)	0.875	24 (3.91±0.96)	**0.001**
***XPA*** ** 5′UTR**				
GG	64 (3.28±0.76)	Ref	51 (3.43±0.83)	Ref
GA	119 (3.37±0.88)	0.527	127 (3.42±0.80)	0.952
AA	49 (3.55±0.92)	0.115	55 (3.36±0.86)	0.626
***XPD*** ** Lys751Gln**				
GG	197 (3.30±0.82)	Ref	203 (3.50±0.79)	Ref
GA + AA	35 (3.29±0.85)	0.961	30 (3.45±0.92)	0.732
***ERCC1*** ** 3′UTR**				
GG	102 (3.33±0.87)	Ref	107 (3.37±0.82)	Ref
GA	110 (3.43±0.75)	0.396	102 (3.43±0.72)	0.636
AA	20 (3.37±0.89)	0.875	24 (3.57±0.91)	0.332
***XPF*** ** Ser835Ser**				
CC	126 (3.31±0.86)	Ref	122 (3.42±0.78)	Ref
CT	87 (3.31±0.76)	0.982	95 (3.57±0.80)	0.163
TT	19 (3.15±0.86)	0.433	16 (3.63±0.97)	0.329

a natural log-transformed.

b Two-sided χ^2^ test.

c Adjusted for consumption of charcoaled food.

## Discussion

Increasing attentions have recently focused on genetic polymorphisms that could modulate human responses to genotoxic insults [18], [19]. However, whether genetic polymorphisms influence the susceptibility of human sperm DNA to toxicant-induced damage is largely unknown. The present study focused specifically on the effects of common DNA repair genetic polymorphisms on PAH-DNA adducts in sperm.

We observed a significant association between *XRCC1* 5′UTR -T/C, *XRCC1* Arg194Trp, and *XRCC1* Arg399Gln polymorphisms and the PAH-DNA adduct formation in sperm. It is notable because *XRCC1* polymorphisms may modify sperm PAH-DNA adducts levels, and hence, may be a useful biomarker to identify individuals susceptible to sperm DNA damage resulting from PAHs exposure. Moreover, the adverse effects of *XRCC1* Arg194Trp and *XRCC1* Arg399Gln were detected only among high PAHs exposure subjects. Similar to our previous studies, we observed a joint effect between *XRCC1* Arg399Gln and PAHs exposure on the susceptibility to sperm DNA fragmentation and male infertility [20]. These findings provided a gene-environment interaction between PAHs exposure and *XRCC1* genetic polymorphisms, and might be of concern owing to the ubiquitous exposure among the non-occupational population.

*XRCC1* encodes an important BER pathway protein that is essential in drawing different components of BER to the sites of DNA damage and promoting repair efficiency [21]. In rodents and primates, the expression of *XRCC1* gene is significantly higher in testis than other tissues, implicating a role in the maintenance of genetic integrity during spermatogenesis [22], [23]. Four functional polymorphisms T-77C, Arg194Trp, Arg280His and Arg399Gln, have been shown to alter DNA repair capacity (DRC) in some phenotypic studies, supporting the association of *XRCC1* SNPs with PAH-DNA adduct levels. Consistent with our study, several previous studies report that the variant alleles of *XRCC1* Arg194Trp and *XRCC1* Arg399Gln are associated with a higher level of DNA adducts in lymphocytes or lung tissue [24]–[26]. However, the difference between sperm and lymphocytes should be noted. Different from the somatic cell, mature sperm cells (spermatids and spermatozoa) have minimal DNA repair capacity, once their chromatin has condensed. Thus the formation of adducts during the late stage of spermatogenesis would not be repaired, leaving the possibility of accumulation of adducts during spermatogenesis. Contrary to expectation, Horak et al [14] observe significantly lower levels of bulky DNA adducts in sperm than those in lymphocytes. It has been proposed that the blood-testis barrier, compact nature of sperm chromatin and effective repair of DNA damage during early stages of spermatogenesis may be the possible mechanisms.

Tobacco smoking and dietary habits with the frequent consumption of charcoal-grilled food are the most important non-occupational exposures to PAHs [27]. In the present study, we found that the consumption of PAH-rich meals at least three times a week contributed significantly to an increase in PAH-DNA adduct formation, consistent with previous reports [28], [29]. However, we did not find any correlation between cigarette smoking and PAH-DNA adducts in spermatozoa. In agreement with the present results, a recent study using immunofluorescent assay also found no relationship between smoking and sperm PAH-DNA adducts (16). One reason could be the misclassification of exposure status, since we did not have an objective measure of smoking, such as cotinine levels. By the determination of cotinine levels in seminal plasma or urine (as an internal dosimeter of cigarette smoke), significant positive associations between PAH-DNA adducts and cotinine levels are found [12], [26], [30]. It is also possible that the benzo(a)pyrene from tobacco smoke does not reach the target tissue sufficiently to produce detectable adduct levels in sperm cells. Thus, it is suggested that sperm cells may not be as sensitive to the effects of smoking as other tissues [31], [32].

The strengths of present study include its high participation rate (>95%), and both genotyping and internal biological markers of exposure available. In particular, we use a sensitive biomarker, the urinary 1-OHP, to assess individual PAHs exposure levels. In addition, urinary metabolites tests can account for the majority of exposure sources and routes. There are also several potential limitations to our study. First, men who visit hospitals may differ from those in general population, and therefore be more “susceptible” to PAHs with a possibility of reporting bias. Second, we only considered those functional SNPs based on prior knowledge, rather than a comprehensive tagging SNP-based approach that would capture most of genetic variations in each gene. Therefore, we cannot discard a potential role of those DNA repair genes in which we did not find associations.

In conclusion, this is the first epidemiologic study to explore the effects of PAHs exposure at general population levels and DNA repair polymorphisms, alone or combined, on sperm PAH-DNA adducts. These observations support the notion of combined influences of genetic susceptibility and environmental exposure on male reproductive parameters. A more comprehensive genetic approach in a larger study population is valuable for further studies.

## Materials and Methods

### Study subjects and sample collection

Study subjects were diagnosed with unexplained male factor infertility from affiliated hospitals of Nanjing Medical University between March 2004 and October 2007 (NJMU Infertile Study). The protocol and consent form were approved by the Institutional Review Board of Nanjing Medical University prior to the study. All activities involving human subjects were done under full compliance with government policies and the Helsinki Declaration. Consecutive eligible men (with wives not diagnosed as infertile) were recruited to participate. There were no significant differences in sampling numbers among years and seasons. After the study procedures were explained and all questions were answered, subjects signed informed consent forms. All participants completed an informed consent and a questionnaire including detailed information, such as age, cigarette smoking, alcohol drinking, dietary habits, residence and other activities that might be expected to influence PAHs exposure. Each subject donated 5 ml of blood for genomic DNA extraction and a single spot urine sample was collected from each subject on the same day as the semen sample. Urine samples were frozen at −20°C until analyses for PAHs metabolites. Totally, 465 subjects whose genotyping data, PAH metabolites and PAH-DNA adducts measures were available were included in this study.

### PAH-DNA adducts determination: Immunofluorescence staining

PAH-DNA adducts were measured by an indirect immunofluorescence method using BPDE-DNA (5D11) monoclonal antibody (sc-52625; Santa Cruz Biotechnology, Santa Cruz CA), with FITC-conjugated secondary antibody. Although, 5D11 recognizes BPDE-DNA adducts, it also cross-reacts with other structurally related PAHs in various affinities; hence the terminology PAH-DNA is commonly used [33]–[36]. The intensity of FITC fluorescence was then detected by the FACSCalibur flow cytometer (BD Biosciences Pharmingen).

In brief, washed sperm were resuspended in 2% paraformaldehyde for 30 min at room temperature. After washing with PBS, samples were resuspended in permeabilization solution (0.2% Triton X-100, 0.1% sodium citrate) for 10 min on ice. Samples were washed twice with 1×PBS, treated with 100 µg/ml RNase at 37°C for 1 h, then with proteinase K (10 µg/ml in at room temperature) for 10 min. To denature the DNA, samples were incubated with 4N HCl for 10 min followed by 50 mM Tris-base (5 min at room temperature). After blocking with 5% normal goat serum in Tris buffer (45 min at 37°C), samples were then incubated with mouse monoclonal antibody raised against BPDE-I-G (dilution 1∶50 in PBS; overnight at 4°C). After washing with PBS, samples were incubated with fluorescein isothiocyanate (FITC)-conjugated goat anti-mouse IgG (dilution 1∶200 in PBS) at 37°C for 45 min. Samples were washed with PBS, and then analyzed immediately by flow cytometry. We analysed 10,000 individual sperm per sample for FITC fluorescence emissions. Mean fluorescence intensity (MFI) was calculated on a logarithmic scale. For each batch, a negative control with normal mouse IgG instead of the 5D11 was performed.

To assess the reliability and concordance of flow cytometry and fluorescence microscopy, 46 semen samples were analyzed simultaneously with both techniques. The immunofluorescence microscopy used for the analysis of DNA adducts was carried out as described previously [16]. Slides were examined with a confocal laser-scanning microscope LSM 710 (Carl Zeiss, Germany). The quantitative analysis of the average fluorescent intensity was measured directly in five fields for each slide by LSM 710 ZEN software (Carl Zeiss) and the mean value was calculated.

### Urinary PAHs metabolites measurement

The urine 1-OHP, as a sensitive PAHs-exposure biomarker, was determined by LC-MS/MS as previously described [6]. The analytes underwent hydrolysis using β-glucuronidase/arylsulfatase (98%, Sigma-Aldrich, England) and separated from the matrix by solid-phase extraction. Then, the metabolites were detected by LC-MS/MS. The limit of detection for 1-OHP was 0.15 µg/l. Creatinine (CR) concentrations were used to adjust PAHs concentrations for variable urine dilution in spot samples. Samples with CR concentrations >300 or <30 mg/dl were considered too concentrated or too dilute to provide valid results and were excluded from the primary analysis.

### Genotyping

DNA was extracted from peripheral blood lymphocytes by standard methods, resuspended in Tris-EDTA buffer (10 mM Tris, 1 mM EDTA), and frozen until use. We genotyped the following SNPs: *XRCC1* 5′UTR -T/C (rs3213245), *XRCC1* Arg194Trp (rs1799782), *XRCC1* Arg280His (rs25489), *XRCC1* Arg399Gln (rs25487), *XPA* 5′UTR -C/T (rs1800975), *XPD* Asp312Asn (rs1799793), *ERCC1* 3′UTR G/T (rs3212986), and *XPF* Ser835Ser (rs1799801). Genotype analysis of all SNPs was done using polymerase chain reaction (PCR) and restriction fragment length polymorphisms (RFLP) techniques. Genotypes of *XRCC1*, *XPA* 5′UTR, *ERCC1* 3′UTR and *XPD* Lys751Gln were determined as described previously [37], [38]. *XPF* Ser835Ser polymorphism was detected using the following primers: 5′-CTGAAACAAAGCAAGCCACA-3′ (forward) and 5′- GACAGGGCTGCTAATTCTGC-3′ (reverse). The 194 bp PCR product was digested overnight by Cail enzyme (MBI Fermentas) and obtained the A/A (194 bp), A/G (194, 130, 64 bp) and G/G (130, 64 bp) genotypes. For quality control, 10% of the samples were randomly genotyped again, and the reproducibility was 100%.

### Statistical analysis

The statistical analyses were performed with the using the Stata statistical package (Version 7.0; StataCorp, LP, USA). All tests were two-sided and the significance level was set at *P*<0.05. Sperm PAH-DNA adducts was presented as mean ± standard deviations (SD). For sperm PAH-DNA adducts and urinary 1-OHP levels were non-normally distributed (checked by skewness-kurtosis tests), these two variables were transformed to achieve normal distributions using the lnskew0 function (natural log transformed). The effects of selected individual characteristics on sperm PAH-DNA adducts were analyzed by *t* test. A Scatter plot and Pearson correlation analysis were conducted to evaluate the relationship between urinary 1-OHP levels and PAH-DNA adducts. Multiple linear regression analysis was applied for the comparison of PAH-DNA adducts as considered for the genotypes of each SNP. Age, smoking status, drinking status, area of residence and habitual consumption of charcoaled food were considered as potential confounders. These selected individual characteristics were included in the final models if they showed any association (*P*<0.20) with sperm PAH-DNA adducts.

## Supporting Information

Figure S1 Distributions of sperm PAH-DNA adducts in 465 ejaculates. (A), adduct values without logarithmic transformation. (B), adduct values underwent natural logarithmic transformation.
(0.40 MB TIF)

Figure S2 Distribution of urine CR-adjusted 1-hydroxypyrene 1-OHP levels in 465 patients. (A), 1-OHP values without natural logarithmic transformation. (B), 1-OHP values underwent natural logarithmic transformation.
(0.41 MB TIF)
