# Supplementary figures and images for: Interactions between Exposure to Environmental Polycyclic Aromatic Hydrocarbons and DNA Repair Gene Polymorphisms on Bulky DNA Adducts in Human Sperm
Source: PLoS One. 2010 Oct 5;5(10):e13145. doi: 10.1371/journal.pone.0013145 (PMC2950145; doi:10.1371/journal.pone.0013145)

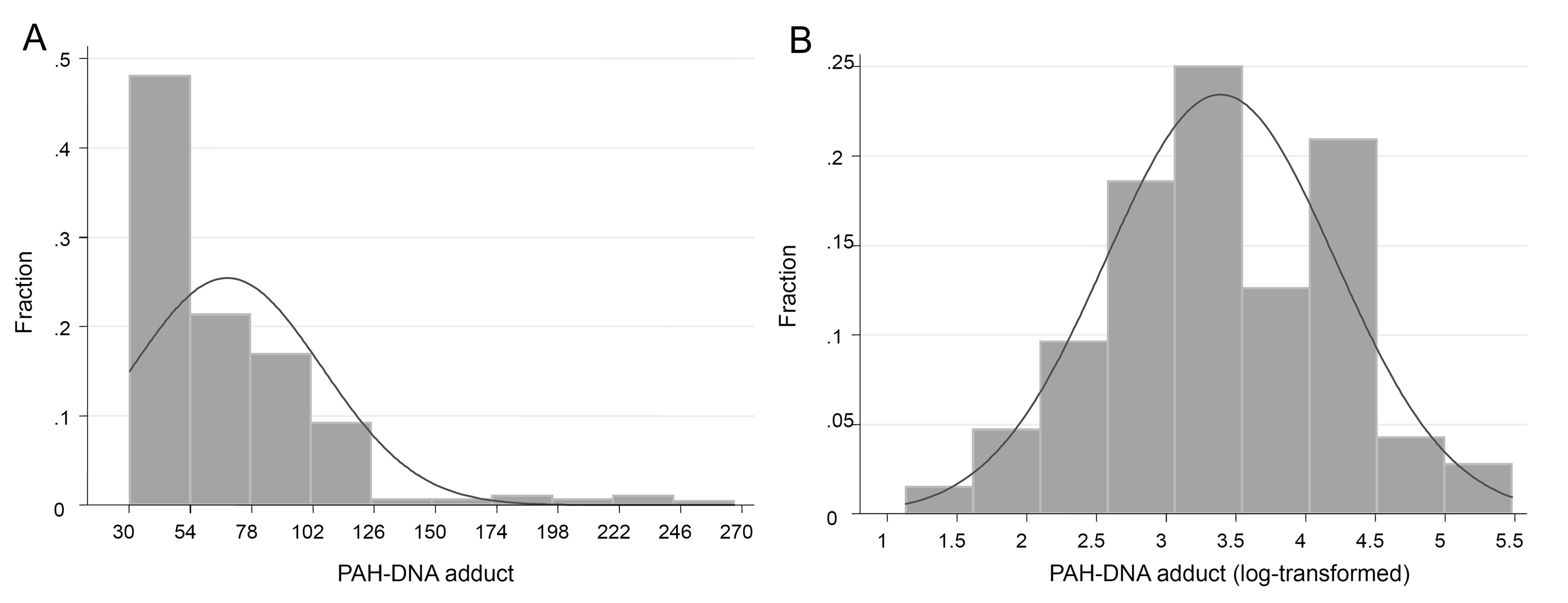

Supplement: Figure S1 — Distributions of sperm PAH-DNA adducts in 465 ejaculates. (A), adduct values without logarithmic transformation. (B), adduct values underwent natural logarithmic transformation. (0.40 MB TIF) [file pone.0013145.s001.tif]

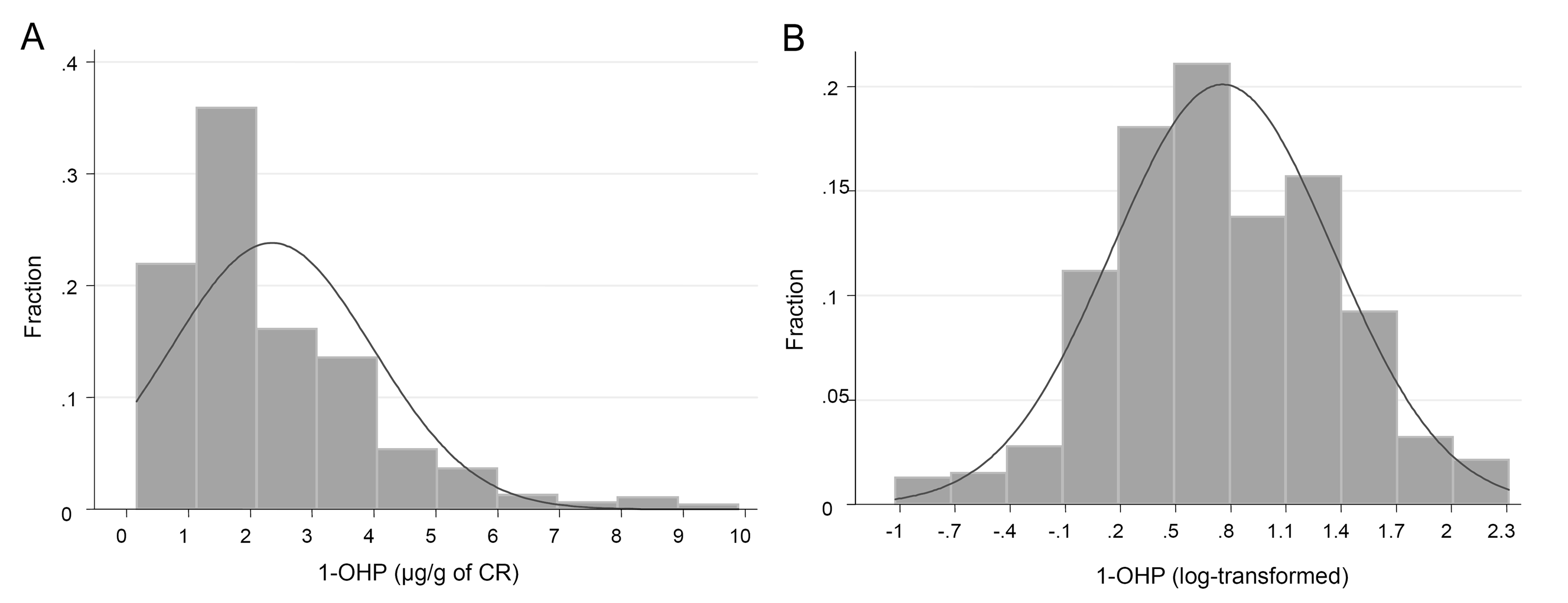

Supplement: Figure S2 — Distribution of urine CR-adjusted 1-hydroxypyrene 1-OHP levels in 465 patients. (A), 1-OHP values without natural logarithmic transformation. (B), 1-OHP values underwent natural logarithmic transformation. (0.41 MB TIF) [file pone.0013145.s002.tif]
